# Readiness for climate change mitigation among anesthesiologists: A before and after study at three German university hospitals

**DOI:** 10.1007/s00101-025-01590-x

**Published:** 2025-09-09

**Authors:** A. A. W. Baumann, L. Grüßer, T. Dölker, F. Lehmann, A. Kowark, S. Ziemann, D. P. Obert, J. Schuler, M. Sander, G. Schneider, C. M. Schulz, F. Schneider, N. Conway

**Affiliations:** 1 TUM School of Medicine and Health, Klinikum rechts der Isar, Department of Anesthesiology and Intensive Care, Technical University of Munich, Ismaninger Str. 22, 81675 Munich, Germany; 2 RWTH Aachen, Aachen University Hospital, Department of Anesthesiology, Aachen, Germany; 3 University Hospital of Giessen, Department of Anesthesiology and Intensive Care Medicine, University of Giessen and Marburg, Giessen, Germany; 4 Klinik für Anästhesie, operative Intensivmedizin und Schmerztherapie, Krankenhaus im Friedrichshain, Berlin, Germany; 5 University Hospital Bonn, Clinic of Anaesthesiology and Intensive Care, University of Bonn, Bonn, Germany; 6 KLUG – Deutsche Allianz Klimawandel und Gesundheit e. V., Berlin, Germany; 7 Department of Anaesthesiology and Intensive Care Medicine, Klinikum Traunstein, Traunstein, Germany

**Keywords:** Organizational readiness, Provider education, Fresh gas flow, Sustainability, Anesthesia, Organisatorische Bereitschaft, Schulung von Behandelnden, Frischgasfluss, Nachhaltigkeit, Anästhesie

## Abstract

**Background:**

Medical societies around the world are exploring strategies to reduce their carbon footprint. In this context, organizational readiness can serve as an important facilitator for the success of change. In this study we assessed whether a series of educational interventions improved anesthesia departments’ organizational readiness for climate change mitigation.

**Methods:**

Anesthesiologists at three German university hospitals were asked to complete a survey on their departments’ organizational readiness for climate change mitigation before and after an educational intervention bundle featuring lectures, posters and stickers was conducted. The second survey included additional questions about the use of climate-friendly low-flow and minimal-flow techniques.

**Results:**

A total of 422 questionnaires were completed, 256 of them prior to the interventions. Most participants noticed the interventions and mostly rated them as “good” or “rather good”. We found high overall levels of organizational readiness. Agreement to statements in the subcategories of cultural and staff readiness increased from a low baseline level. Participants reported an increased use of minimal-flow techniques (51.6% vs. 66.3% endotracheal tube) and of low-flow techniques (41.0% vs. 57.8% laryngeal mask) during inhalational anesthesia.

**Conclusion:**

Following our educational intervention bundle, organizational readiness at the participating institutions increased and a reduction in consumption of volatile anesthetics was reported. Pending proof of causality, these results encourage further exploration and the application of educational interventions on climate change mitigation in anesthesiology.

**Graphic abstract:**

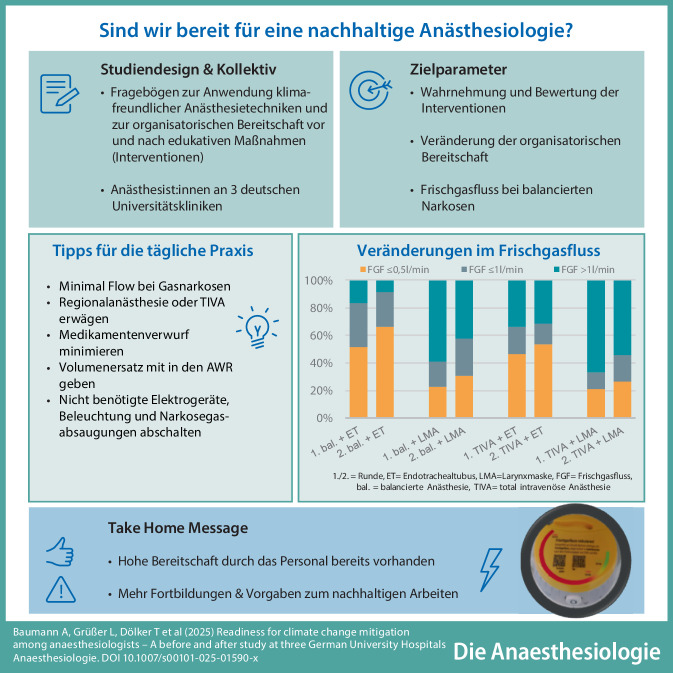

**Supplementary Information:**

The online version of this article (10.1007/s00101-025-01590-x) contains the questionnaire, the detailed results, and an overview of the interventions. Please scan the QR code.

## Abstract (de)

**Hintergrund:**

Angesichts der sich verschärfenden Klimakrise und ihrer erheblichen gesundheitlichen Folgen suchen medizinische Fachgesellschaften weltweit nach Wegen hin zu einer nachhaltigen Gesundheitsversorgung. Ein entscheidender Faktor für den Erfolg von Veränderungen kann dabei die organisatorische Bereitschaft sein. Die vorliegende Studie prüft, ob gezielte themenbezogene Interventionen diese Bereitschaft im Hinblick auf Klimaschutzmaßnahmen steigern konnten.

**Methoden:**

An drei deutschen Universitätskliniken wurden ärztliche Mitarbeitende der Anästhesieabteilungen gebeten, vor und nach der Durchführung von Fortbildungsmaßnahmen – bestehend aus Vorträgen, Postern und Aufklebern – eine standardisierte Umfrage zur organisatorischen Bereitschaft ihrer Abteilungen für Klimaschutzmaßnahmen auszufüllen. Zusätzlich wurden Fragen zur Anwendung klimafreundlicher Low-Flow- und Minimal-Flow-Techniken gestellt.

**Ergebnisse:**

Insgesamt wurden 422 Fragebögen ausgewertet, davon 256 vor und 166 nach Durchführung der Interventionen. Die Interventionen wurden von den Teilnehmenden wahrgenommen und überwiegend als „gut“ oder „eher gut“ bewertet. Bei insgesamt hoher organisatorischer Bereitschaft zu beiden Messzeitpunkten zeigte sich nach Durchführung der Interventionen eine deutliche Steigerung der Unterkategorien *kulturelle* und *personelle Bereitschaft*. Zudem gaben die Befragten an, bei balancierter Anästhesie häufiger Minimal-Flow-Techniken (51,6 % vs. 66,3 % unter Verwendung eines Endotrachealtubus) und Low-Flow-Techniken (41,0 % vs. 57,8 % unter Verwendung einer Larynxmaske) anzuwenden, als vor Durchführung der Interventionen.

**Schlussfolgerung:**

Die Ergebnisse deuten darauf hin, dass gezielte Fortbildungsmaßnahmen zur Steigerung der organisatorischen Bereitschaft für Klimaschutzmaßnahmen in der Anästhesiologie beitragen und den Einsatz klimafreundlicher Anästhesietechniken fördern können. Auch wenn ein direkter Kausalzusammenhang nicht belegt werden konnte, unterstreichen die Befunde die Notwendigkeit, solche Bildungsmaßnahmen weiterzuentwickeln und breit in der klinischen Praxis zu etablieren, um klimaschädliche Emissionen in der Anästhesiologie zu reduzieren.

## Treten Sie in den Austausch

Diese Arbeit wurde für *Die Anaesthesiologie* in Englisch eingereicht und angenommen. Die deutsche Zusammenfassung wurde daher etwas ausführlicher gestaltet. Wenn Sie über diese Zusammenfassung hinaus Fragen haben und mehr wissen wollen, nehmen Sie gern in Deutsch über die Korrespondenzadresse am Ende des Beitrags Kontakt auf. Die Autorinnen und Autoren freuen sich auf den Austausch mit Ihnen.

## Brief introduction

The climate crisis threatens public health, while the healthcare sector itself significantly contributes to the problem. Fundamental transformation is needed for effective climate adaptation and mitigation. Especially anesthesia can reduce the environmental impact without compromising quality of care. Studies suggest that training, guidelines and incentives can support mitigation. Change management theory highlights organizational readiness as the key to successful change. We assessed this readiness and the impact of educational interventions among anesthesiologists at three German university hospitals.

## Introduction

The rapidly progressing climate crisis poses an increasing and unprecedented global threat to well-being, health and survival [1]. Paradoxically, the healthcare sector itself significantly contributes to the climate crisis. It must therefore undergo fundamental transformations towards effective climate adaptation and mitigation [2–4]. This leaves medical societies to search for ways of providing healthcare without causing harm [5, 6].

Anesthesia plays a particularly important role here, as many anesthesia practices, such as equipment and facility design choices can substantially decrease environmental impact, without compromising the quality of medical care [6–8]. Current studies suggest that climate change mitigation can be effectively supported through a combination of training programs, clear guidelines and structural incentives [9–11]. Change management theory emphasizes organizational readiness as a critical prerequisite for successfully implementing change initiatives [12]. Therefore, research on anesthesia departments’ organizational readiness for climate change mitigation is needed.

On the basis of this background, we conducted a multicenter study among anesthesiologists from three German university hospitals. The aim was to assess their departments’ organizational readiness for climate change mitigation and to explore whether it could be increased by targeted educational interventions. Additionally, the study aimed at promoting climate-friendly anesthesia practices. The results of this investigation are intended to support the transformation towards environmentally sustainable anesthesia.

## Methods

### Study setting and participants

This multicenter observational before and after study was conducted at three German university hospitals. It consisted of an online questionnaire and educational interventions. The questionnaire was answered before (survey I) and in slightly modified form, after the interventions had taken place (survey II).

Starting in December 2020, anesthesiologists were invited to participate in an online survey (PEEP, Provider Education and Evaluation Project) which addressed, among other topics, departments’ organizational readiness for climate change mitigation [13]. For both surveys, recruitment consisted of an email to all anesthesiologists employed at the three departments, followed by reminder emails. Due to different ethical approval timelines and staffing schedules, the online survey was conducted at slightly different times and was closed after 4 weeks in each of the 3 departments. Subsequently, the participating study centers launched interventions aimed at reducing greenhouse gas emissions and raising awareness for climate action, as described in more detail below.

Survey II was carried out approximately 1 year after survey I. The study period ended in July 2022. In addition, the respective departments conducted single-center sub-studies exploring different areas of climate-friendly behaviours in anesthesia in more detail. Several of these studies have already been published [10, 11, 13]. Each of the three university hospitals’ ethics committees approved the study (632/20 S for the coordinating center in Munich, Ethikkommission der Technischen Universität München). Informed consent from participants was obtained prior to their participation in each survey. The online survey tool unipark.com (EFS survey by Tivian, Tivian XI GmbH, Cologne, Germany) enabled anonymous data collection. A more detailed description of the study setting, including the validation and development of the questionnaire, can be found in a separate publication on survey I in Munich [13].

### Interventions

As described elsewhere [11], several educational interventions were implemented across all three anesthesiology departments and were slightly adapted to different local circumstances. As reported in the literature [9], stickers highlighting the importance of reducing fresh gas flow (FGF) in inhalational anesthesia cases were placed on anesthesia machines and vaporizers. They also displayed QR codes linked to (a) the position paper on ecological sustainability of the German Society of Anaesthesiology and Intensive Care Medicine (DGAI) and the Professional Association of German Anaesthesiologists (BDA) [17] and (b) a mobile phone application for calculating the climate impact of anesthesia based on any given ventilator setting [18].

Additional stickers were placed on computers and light switches that were not part of emergency equipment. They displayed reminders to act environmentally friendly by turning the respective lights and devices off when not in use. Lectures informed about the environmental impact of anesthesiology, suggesting adding several easy to implement, climate-friendly actions to anesthesiologists’ daily clinical routine. This information was likewise presented on posters placed in locations frequented by anesthesia staff (e.g., postanesthesia care unit, examination rooms, dining areas; [see appendix 1]).

### Questionnaire

The questionnaire consisted of a total of 65 items. These were divided into 6 areas of interest:

1. Personal information (4 items)—corresponding to “demographics”.
2. Personal attitudes (9 items)—corresponding to “psychological readiness”.
3. Current research and knowledge (3 items)—corresponding to “knowledge” (including question on “environmental impact estimation”).
4. Readiness for change (13 items)—corresponding to “structural readiness”.
5. Potential opportunities (24 items, e.g. rating of the benefits of climate-friendly measures).
6. Practical considerations (12 items, e.g. preferred fresh gas flow (FGF) settings).

Except for two open text questions, all questions were forced choice, using a 4-point Likert scale. As described in more detail before [13], items were partially inspired by pre-existing surveys published by Ard et al., Petre et al. and McGain et al., [14–16], as well as the framework on organizational readiness described by Shahrasbi and Paré [12]. The questionnaire was checked for comprehensibility and congruence, although no formal preliminary testing was performed [13].

In survey II we added four questions asking for feedback on the interventions. The survey tool only allowed respondents to continue and complete the survey once all previous items had been answered (Supplement 1. Questionnaire).

### Outcome variables

**Fig. 1:**
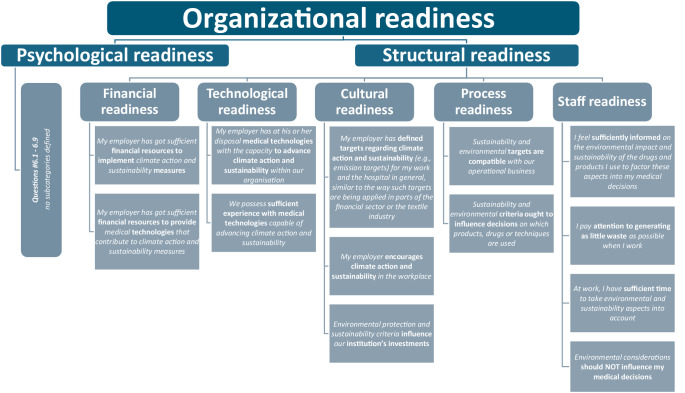
Categories and subcategories with corresponding questions of organizational readiness based on the framework by Shahrasbi and Paré

We defined the following outcome variables:

1. Awareness and evaluation of the interventions
  awareness: percentage of respondents who noticed each intervention (training, stickers, posters)
  evaluation: percentage of positive ratings for each intervention.
2. Organizational readiness for change (percentage of agreement with corresponding statements) (Fig. 1)
  psychological readiness
  structural readiness (subcategories: financial, technological, cultural, process and staff readiness).
3. Adjustment in daily practice: percentage of participants reporting the usage of settings in line with low-flow or minimal-flow anesthesia when applying volatile anesthetics
  in cases with endotracheal tubes
  in cases with laryngeal mask airway devices.

Due to the length of the questionnaire and the various topics covered, in-depth analysis of all items would have exceeded the scope of this study. Therefore, we focused our attention on organizational readiness as it is an essential and poorly understood prerequisite for climate change mitigation.

### Statistical analysis

As the results for all variables are presented using a categorial scale, descriptive statistics are presented as whole numbers, followed by percentages in brackets. Exploratory analysis was conducted using Mann-Whitney‑U tests and χ^2^-tests to compare non-parametric responses from surveys I and II. Additional, exploratory subgroup analyses were conducted to assess potential associations between demographic and survey responses. The SPSS Statistics (Version 29, IBM Cooperation, Armonk, NY, USA) was used for all statistical analysis, and the threshold for statistical significance was defined as *p* < 0.05.

## Results

A total of 256 questionnaires were completed in survey I (before the interventions) and 166 were completed in survey II (after the interventions). Given that 460 anesthesiologists were invited to participate in survey I and 420 were invited in survey II, this equates to a response rate of 55.7% for survey I and 39.5% for survey II.

### Demographics

Comparative analysis of the demographic data from surveys I and II found no inconsistencies (Table 1).

**Table 1:** Demographic characteristics of the participants from survey I (256 participants) and survey II (166 participants)

Participant demographics		Survey I	Survey II
Participants	Munich	104 (40.6%)	47 (28.3%)
	Giessen	54 (21.1%)	36 (21.7%)
	Aachen	98 (38.3%)	83 (50.0%)
Age in years	< 30	43 (16.8%)	24 (14.5%)
	30–39	135 (52.7%)	79 (47.6%)
	40–49	52 (20.3%)	43 (25.9%)
	50–59	12 (4.7%)	14 (8.4%)
	≥ 60	14 (5.5%)	6 (3.6%)
Years of experience	< 3	56 (21.9%)	30 (18.1%)
	3–5	70 (27.3%)	40 (24.1%)
	6–10	56 (21.9%)	39 (23.5%)
	11–15	33 (12.9%)	20 (12.0%)
	16–25	27 (10.5%)	26 (15.7%)
	> 25	14 (5.5%)	11 (6.6%)
Current position	Trainee doctor 1st or 2nd year of anesthesia training	46 (18.0%)	22 (13.3%)
	Trainee doctors in their third year of anesthesia training or above	104 (40.6%)	61 (36.7%)
	Board-certified anesthesiologist	55 (21.5%)	47 (28.3%)
	Head of department or consultant	51 (19.9%)	36 (21.7%)
Children	Yes	111 (43.4%)	78 (47.0%)
	None	145 (56.6%)	88 (53.0%)

### Awareness and evaluation of the interventions

**Fig. 2:**
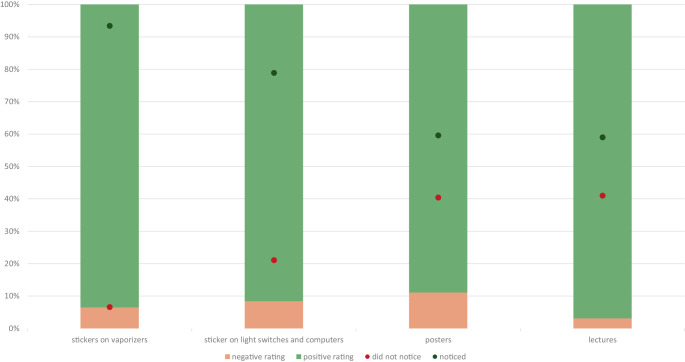
Interventions-awareness in percentage of participants and evaluation in percentage of those, who noticed the educational interventions

Overall, most participants noticed at least one of the interventions, and all interventions received mostly positive ratings (Fig. 2).

### Organizational readiness

#### Psychological readiness

**Fig. 3:**
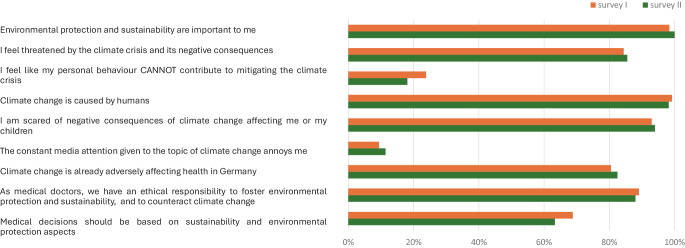
Psychological readiness: percentage of agreement (“very good” and “good”) to statements numbers 6.1–6.9 in survey I and survey II

Participants indicated a high level of agreement to seven out of nine statements on psychological readiness, and two statements were agreed to by distinctly fewer participants. No statistically significant differences in agreement between survey I and survey II were found (Fig. 3).

#### Structural readiness

**Fig. 4:**
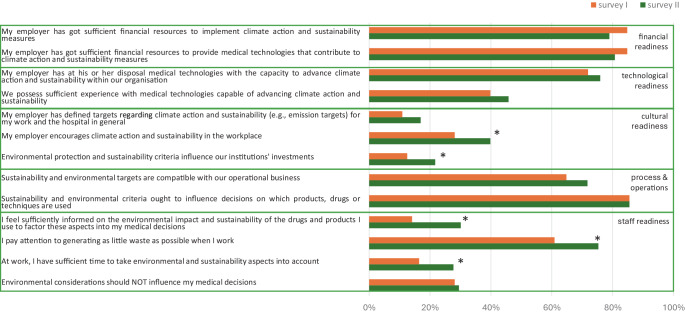
Structural readiness: percentage of agreement (“very good” and “good”) to statements numbers 10.1–10.7 and 11.1–11.6 in survey I and survey II. *Asterisk* statistically significant improvement in survey II

Most statements related to financial, technological, and process readiness received consistently high agreement rates across surveys I and II. In contrast, most statements on cultural and individual readiness showed a statistically significant increase in agreement in survey II, starting from comparatively low baseline values in survey I. In summary, overall structural readiness was high from the start, with a gap in the subcategories of cultural und staff readiness. These subcategories showed improvement after the interventions (Fig. 4).

### Adjustment in anesthesia practice

**Fig. 5:**
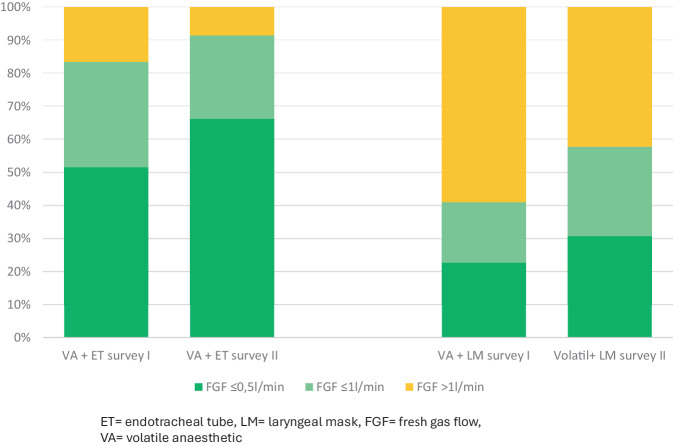
Percentage of participants reporting to use settings in line with low-flow or minimal-flow anesthesia when applying volatile anesthetics. *ET* endotracheal tube, *LM* laryngeal mask, *FGF* fresh gas flow, *VA* volatile anaesthetic

When asked to state their preferred FGF rates for cases involving volatile anesthetics, the average response was markedly higher when respondents were asked to assume a laryngeal mask airway device was in place, as opposed to an endotracheal tube. This was true for both surveys I and II. In survey II, the average preferred FGF rate in cases involving volatile anesthetics was significantly lower than in survey I for either type of airway device (Fig. 5).

Exploratory analyses did not reveal consistent differences between subgroups, such as current professional positions. Due to the exploratory nature of these analyses and limitations in data quality, detailed results are not presented. All other results can be found in supplement 2.

## Discussion

To the best of our knowledge, this is the first observational study on organizational readiness for climate change mitigation among anesthesiologists. The educational interventions were noticed by most of the participants and rated positively. We found that overall organizational readiness was high to start with. Cultural and staff readiness were initially low but showed improvement after the interventions. The reported use of climate-friendly minimal-flow and low-flow techniques in volatile anesthesia substantially increased after the interventions.

Understanding organizational readiness may be very helpful for the successful implementation of change [12]. To determine whether the interventions had an impact on the organizational readiness of the three departments studied, we first investigated if the interventions were noticed and how they were evaluated. In accordance with previous research on similar measures [9], stickers on anesthesia devices detailing the climate impacts of volatile anesthesia, and dedicated lectures on how to implement climate change mitigation in daily practice were noteworthily popular among a list of well-received interventions.

To investigate organizational readiness, a total of 9 questions on psychological readiness and 13 questions on structural readiness were asked, based on the framework published by Shahrasbi and Paré [12]. It is important to note that this framework assesses perceived organizational readiness rather than verifiable institutional capacities. Accordingly, the aim was not to effect or measure organizational change but to examine whether educational interventions could shift the subjective perception of these conditions. As previously reported for a subset of the study population [13], we found high agreement rates to the statements assessing psychological readiness, but limitations within structural readiness, particularly in the subcategories cultural and staff readiness. Cultural readiness, in particular, has been identified as a key factor influencing recycling behavior [19], while shortcomings in this area have been cited as a major barrier to environmental sustainability efforts [13, 15]. The discrepancy between low cultural readiness and a high perceived availability of financial and technical resources may reflect a gap between the availability of resources and their practical application through institutional leadership, potentially hindering effective climate measures and requiring further attention.

At the same time, it is all the more noteworthy that both cultural readiness and staff readiness showed substantial improvement after the interventions, suggesting that the gap in structural readiness was successfully narrowed. Combined with the already high levels of psychological readiness, this is an important improvement in overall organizational readiness for climate change mitigation.

Reducing FGF and using minimal-flow is advertised as safe by manufacturers and recommended by professional anesthesia societies around the globe [6, 8, 20]. To explore whether the interventions were able to influence not only organizational readiness but also the use of these climate-friendly techniques, the respondents were asked about their usual FGF settings during steady-state volatile anesthesia. Contrary to current recommendations, the reported use of minimal-flow especially while using laryngeal mask airway devices was very low, suggesting a substantial gap of knowledge regarding best practice and safe procedures. Supported by findings from the sub-studies in Aachen and Giessen linking the interventions to measurable reductions in anesthetic gas consumption [10, 11], increased use of minimal-flow and low-flow volatile anesthesia was reported after the interventions. It therefore appears possible that the interventions may not only have helped establish recommended anesthesia practices but also helped reduce environmental impact and costs.

As well as positive changes in participants’ clinical practice and organizational readiness, we observed a shift in the way they perceived their employers’ support and engagement for climate change mitigation. Taken together with the fact that respondents felt better informed and more able to reduce waste in their workplace, the case for low-cost interventions promoting climate change mitigation in anesthesia appears strong. Ergo, while top-down implementation of climate change mitigation in the healthcare sector remains urgently needed, its success can be significantly enhanced through agenda setting and training programs, both increasing the awareness of the issue and providing concrete options for action.

## Limitations

Causality between the educational interventions and the survey outcomes cannot be established due to the lack of a control group and other unmeasured confounding factors, such as the influence of public discourse on climate change or the global increase in climate-related disasters. Additionally, social desirability bias and sample bias such as self-selection, sector and regional bias are common limitations associated with survey-based research, limiting the generalizability of our findings. Slight interdepartmental differences in the interventions may have occurred due to varying local circumstances. Overall, participation in survey II was not as high as expected, which appears likely to be a consequence of the length of the questionnaire.

To better validate the interventions and achieve a measurable, positive impact on climate action in anesthesiology, future research could benefit from a larger, more representative sample and a shorter questionnaire.

## Conclusion

Our study highlights the importance of organizational readiness for climate change mitigation within anesthesia departments. While overall organigational readiness was already high when the first survey was conducted, notable deficits were identified in the subcategories cultural and staff readiness; however, following the interventions, these subcategories experienced substantial improvements. Respondents also reported lowering the average FGF rates during anesthesia. Despite certain study limitations, our findings suggest that targeted, straightforward and low-cost interventions, as used in this study, can have a measurable, positive impact on climate change mitigation. Future research should aim for larger, more representative samples to validate these results and further assess the impact on climate action within the field.

## Supplementary Information

Questionnaire

Results of the survey

Interventions

## Data Availability

The data that support the findings of this study are not openly available due to reasons of sensitivity and are available from the corresponding author upon reasonable request.

## Declarations

For this article no studies with animals were performed by any of the authors. All studies mentioned were in accordance with the ethical standards indicated in each case.
