# Supplementary material for: Readiness for climate change mitigation among anesthesiologists: A before and after study at three German university hospitals
Source: Anaesthesiologie. 2025 Sep 9;74(10):646–54. doi: 10.1007/s00101-025-01590-x (PMC12484283; doi:10.1007/s00101-025-01590-x)
Supplement: Supplementary file 1 — Questionnaire [file 101_2025_1590_MOESM1_ESM.pdf]

Supplementary material for the article "Readiness for climate change mitigation among anesthesiologists. A before and after study at three German University Hospitals" by Baumann, AAW; Grüßer, L; Dölker, T et al. (2025) in *Die Anaesthesiologie*.

The article and supplementary material are available at www.springermedizin.de. Please enter the article title in the search field.

## *Supplement 1: Provider Education and Evaluation Survey.*

- Pseudonym
  1. **Please enter your pseudonym here**
- Personal Information
  2. What age are you?
     a. Under 30
     b. 30 – 39
     c. 40 – 49
     d. 50 – 59
     e. 60 years of age or older

  3. How many years of working experience have you collected so far? (as a medical doctor)
     a. Less than 3 years
     b. 3 – 5 years
     c. 6 – 10 years
     d. 11 – 15 years
     e. 15 - 25 years
     f. Over 25 years

  4. Which of the following best describes your current position?
     a. trainee doctor in first or second year of anesthesia training
     b. trainee doctor in their third year of anesthesia training or above
     c. board-certified anesthetist
     d. head of department or consultant

     *(Of note: "Training" in this context refers to German anesthesia training, which consists of a 5-year traineeship after graduating from medical school. In Germany, board-certified anesthetists may or may not earn a promotion to become consultants, a position that is therefore not achieved automatically by virtue of seniority or experience.)*

  5. Do you have children?
     a. yes
     b. no

- Regarding your personal attitudes

  6. To what degree would you agree to the following statements? (one response per item, taken from the following scale: completely agree – rather agree – rather disagree – completely disagree)

     1. Environmental protection and sustainability are important to me
     2. I feel threatened by the climate crisis and its negative consequences
     3. I feel like my personal behavior CANNOT contribute to mitigating the climate crisis

4. Climate change is caused by humans
5. I am scared of negative consequences of climate change affecting me or my children
6. The constant media attention given to the topic of climate change annoys me
7. Climate change is already adversely affecting health in Germany
8. As medical doctors, we have an ethical responsibility to foster environmental protection and sustainability, and to counteract climate change
9. Medical decisions should also be based on sustainability and environmental protection aspects

- QUESTIONS ON CURRENT RESEARCH AND KNOWLEDGE

7. Which of the following goals is NOT listed as part of the United Nations' 2030 Agenda for Sustainable Development? (Single choice, correct answer marked in green)
    a. climate action
    b. affordable and clean energy
    c. responsible consumption and production
    d. good health and well-being
    e. limiting global heating to 1.5° Celsius

8. To what degree does the health care sector currently contribute to Germany's overall CO2 emissions? (Single choice, correct answer marked in green)
    a. 0,1%
    b. 0,5%
    c. 1%
    d. 5%
    e. 10%

9. German physicians' pension funds oversee funds in excess of 100 billion euro. Which of the following statements regarding sustainable investment is INCORRECT? (Single choice, correct answer marked in green)

    *(Of note: All practicing physicians in Germany are obliged to contribute to their respective regional physicians' pension funds)*

    a. Environmental, social, and governance (ESG) criteria guide investment decisions in order to avoid adverse effects on health
    b. Applying ESG (environmental, social, and governance) criteria is linked to lower returns
    c. An investment's CO2 footprint can be quantified
    d. German physicians' pension funds do not currently offer insights into their investment structure
    e. German physicians' pension funds are managed by physicians

- QUESTIONS ON READINESS FOR CHANGE

10. To what degree do you agree with the following statements regarding yourself and your employer? (Every single statement: completely agree – rather agree – rather disagree – completely disagree)

    *Financial readiness*

    1. My employer has got sufficient financial resources to implement climate action and sustainability measures

2. My employer has got sufficient financial resources to provide medical technologies that contribute to climate action and sustainability measures

*Technological readiness*

3. My employer has at his or her disposal medical technologies with the capacity to advance climate action and sustainability within our organization
4. We possess sufficient experience with medical technologies capable of advancing climate action and sustainability

*Cultural readiness*

5. My employer has defined targets regarding climate action and sustainability (e.g., emission targets) for my work and the hospital in general, similar to the way such targets are being applied in parts of the financial sector or the textile industry
6. My employer encourages climate action and sustainability in the workplace
7. Environmental protection and sustainability criteria influence our institution's investments

11. *To what degree do you agree with the following statements regarding yourself and your employer?*

*Process and operations readiness*

1. Sustainability and environmental targets are compatible with our operational business
2. Sustainability and environmental criteria ought to influence decisions on which products, drugs or techniques are used

*Staff readiness*

3. I feel sufficiently informed on the environmental impact and sustainability of the drugs and products I use to factor these aspects into my medical decisions
4. I pay attention to generating as little waste as possible when I work
5. At work, I have sufficient time to take environmental and sustainability aspects into account
6. Environmental considerations should NOT influence my medical decisions

- POTENTIAL OPPORTUNITIES

12. To what degree would you describe the following measures to reduce a hospital's ecological footprint as reasonable? (one response per item, taken from the following scale: very reasonable – rather reasonable – not very reasonable – not at all reasonable)
    1. Switch computers off at night, or set them to standby mode
    2. Reduce paper use (i.e., by digitalization or two-sided printing of documents)
    3. At night, switch off air conditioning in operation theatres outside of pre-determined emergency response areas
    4. Sterilize and re-use originally single-use products, such as laryngeal masks
    5. Replace conventional lighting with LEDs
    6. Install solar power units for self-sufficient energy production
    7. Build and renovate hospitals to create "green buildings" (e.g., according to the DGNB or BNB standards used in Germany.

*Equivalent standards or ratings systems in the UK or US would be BREEAM and IGCC*)

8. Choose type of volatile anesthetic based on its environmental footprint
9. Recycle volatile anesthetics
10. Use pre-filled syringes (e.g., saline solution; the syringes would NOT be drawn up manually)
11. Use re-useable products
12. Establish packaging-saving processes (for example, pre-pack central venous catheter sets)

13. Which of the following options might help strengthen environmental protection in hospitals? (one response per item, taken from the following scale: very good option – rather good option – rather bad option – very bad option)
    1. Increase efforts to inform and educate employees
    2. Increase efforts to inform and educate decision makers
    3. Establish SOPs (standard operating procedures) for climate action
    4. Integrate the issue into the relevant existing SOPs
    5. Acquire financing, such as institutional grant money
    6. Further research aimed at gauging the usefulness of various environmental protection measures
    7. Generate increased awareness for the subject with
        1. Posters
        2. Presentations
        3. Emails
        4. Podcasts
        5. Workshops

14. Further suggestions (free text)

- PRACTICAL CONSIDERATIONS

15. Please estimate the environmental impact of volatile anesthesia. The environmental impact of one hour of volatile general anesthesia at a fresh gas flow rate of 1.5 l/min is roughly equivalent to traveling what distance with an average modern car at 56 mph? (Single choice, correct answer marked in green)
    a. 280 miles (using desflurane)
    b. 28 miles (using desflurane)
    c. 2.8 miles (using desflurane)
    d. 280 miles (using sevoflurane)
    e. 2.8 miles (using sevoflurane)

16. Which fresh gas flows would you typically select during general anesthesia for the following constellations? (Please insert numbers)
    1. Volatile anesthesia + endotracheal tube
    2. Volatile anesthesia + laryngeal mask
    3. TIVA + endotracheal tube
    4. TIVA + laryngeal mask

17. Please explain to what degree you would consider the following points to be relevant limiting factors preventing you from lowering fresh gas flow in volatile anesthesia (assuming there is no acute, short-term leakage to compensate for, such as accidental disconnection): (Multiple choice)
    a. Maintaining sufficient anesthetic gas supply

b. Rapid wash-in of volatile anesthetic
c. Maintaining sufficient O2 supply
d. Preventing possible negative pressure pulmonary oedema upon return of spontaneous breathing
e. Possible consequences of compound A formation
f. Ventilator model not suitable for lower fresh gas flow

18. Other limiting factors not listed above (Free text)

- ADDITIONAL QUESTIONS IN SURVEY II

19. How would you evaluate the educational interventions on the environmental impact of anesthesiology implemented in your department over the past year? (one response per item, taken from the following scale: good – rather good – rather bad – bad – I did not notice them)
    1. Stickers on anesthesia machines and vaporizers
    2. Stickers on computers and light switches
    3. Posters
    4. Lectures on the environmental impact of anesthesiology
