# Supplementary material for: Readiness for climate change mitigation among anesthesiologists: A before and after study at three German university hospitals
Source: Anaesthesiologie. 2025 Sep 9;74(10):646–54. doi: 10.1007/s00101-025-01590-x (PMC12484283; doi:10.1007/s00101-025-01590-x)
Supplement: Supplementary file 2 — Results of the survey [file 101_2025_1590_MOESM2_ESM.pdf]

Supplementary material for the article "Readiness for climate change mitigation among anesthesiologists. A before and after study at three German University Hospitals" by Baumann, AAW; Grüßer, L; Dölker, T et al. (2025) in *Die Anaesthesiologie*.

The article and supplementary material are available at www.springermedizin.de. Please enter the article title in the search field.

Results of survey I: 256 participants; survey II: 166 participants

1.1. Knowledge

Absolute numbers and percentage of correct answers concerning items on knowledge

	Survey I	Survey II
Goals of the United Nations' 2030 Agenda for Sustainable Development	82 (32,0%)	48 (28,9%)
Current contribution of Germany's health care sector to its overall CO2 emission	138 (53,9%)	107 (64,5%)
Sustainable investment	55 (21,5%)	50 (30,1%)
Estimation of environmental impact of one hour of volatile general anaesthesia	128 (50,0%)	89 (53,6%)

2.1 Potential opportunities to reduce a hospital's ecological footprint

Absolute numbers and percentage of "very reasonable" and "rather reasonable" rating

	Survey I	Survey II
Switch computers off at night, or set them to standby mode	242 (94,9%)	163 (98,2%)
Reduce paper use (i.e., by digitalization or two-sided printing of documents)	252 (98,4%)	162 (97,6%)
At night, switch off air conditioning in operation theatres outside of pre-determined emergency response areas	227 (88,7%)	153 (98,2%)
Sterilize and re-use originally single-use products, such as laryngeal masks	120 (46,9%)	88 (53,0%)
Replace conventional lighting with LEDs	253 (98,8%)	165 (99,4%)
Install solar power units for self-sufficient energy production	249 (97,3%)	162 (97,6%)
Build and renovate hospitals to create "green buildings"	248 (96,9%)	161 (97,0%)
Choose type of volatile anesthetic based on its environmental footprint	220 (85,9%)	147 (88,6%)

Recycle volatile anesthetics	241 (94,5%)	158 (96,3%)
Use pre-filled syringes (e.g., saline solution; the syringes would NOT be drawn up manually)	139 (54,7%)	98 (59,4%)
Use re-useable products	241 (94,5%)	155 (95,1%)
Establish packaging-saving processes (for example, pre-pack central venous catheter sets)	244 (96,1%)	163 (98,2%)

2.2. Potential opportunities to help strengthen environmental protection in hospitals

Absolute numbers and percentage of “very good option” and “rather good option” rating

	Survey I	Survey II
Increase efforts to inform and educate employees	249 (97,3%)	163 (98,2%)
Increase efforts to inform and educate decision makers	252 (98,4%)	165 (99,4%)
Establish SOPs (standard operating procedures) for climate action	239 (93,7%)	158 (95,2%)
Integrate the issue into relevant existing SOPs	243 (95,3%)	159 (95,8%)
Acquire financing, such as institutional grant money	250 (98,0%)	162 (97,6%)
Further research aimed at gauging the usefulness of various environmental protection measures	247 (96,9%)	161 (97,0%)
Generate increased awareness for subject with		
- Posters	197 (77,3%)	131 (78,9%)
- Presentations	228 (89,1%)	152 (91,6%)
- Emails	173 (67,8%)	110 (66,7%)
- Podcasts	178 (70,4%)	107 (64,8%)
- Workshops	247 (97,2%)	159 (97,0%)

3.1 Practical consideration

Absolute numbers and percentage of “very relevant” and “relevant” rating of limiting factors preventing from lowering fresh gas flow in volatile anaesthesia

	Survey I	Survey II
Maintaining sufficient anaesthetic gas supply	38 (50,0%)	41 (53,9%)
Rapid wash-in of volatile anaesthetic	60	55

	(78,9%)	(72,4%)
Maintaining sufficient O2 supply	51 (67,1%)	46 (60,5%)
Preventing possible negative pressure pulmonary oedema upon return of spontaneous breathing	25 (32,9%)	28 (36,8%)
Possible consequences of compound A formation	12 (15,8%)	8 (10,5%)
Ventilator model not suitable for lower fresh gas flow	41 (53,9%)	32 (42,1%)
