# Supplementary material for: Readiness for climate change mitigation among anesthesiologists: A before and after study at three German university hospitals
Source: Anaesthesiologie. 2025 Sep 9;74(10):646–54. doi: 10.1007/s00101-025-01590-x (PMC12484283; doi:10.1007/s00101-025-01590-x)
Supplement: Supplementary file 3 — Interventions [file 101_2025_1590_MOESM3_ESM.pdf]

Supplementary material for the article "Readiness for climate change mitigation among anesthesiologists. A before and after study at three German University Hospitals" by Baumann, AAW; Grüßer, L; Dölker, T et al. (2025) in *Die Anaesthesiologie*.
The article and supplementary material are available at www.springermedizin.de. Please enter the article title in the search field.

Interventions

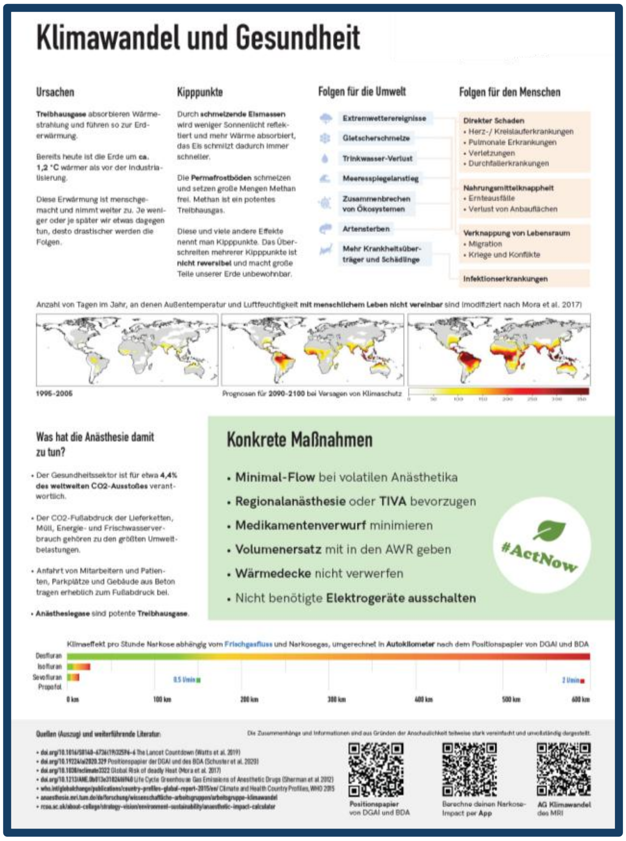

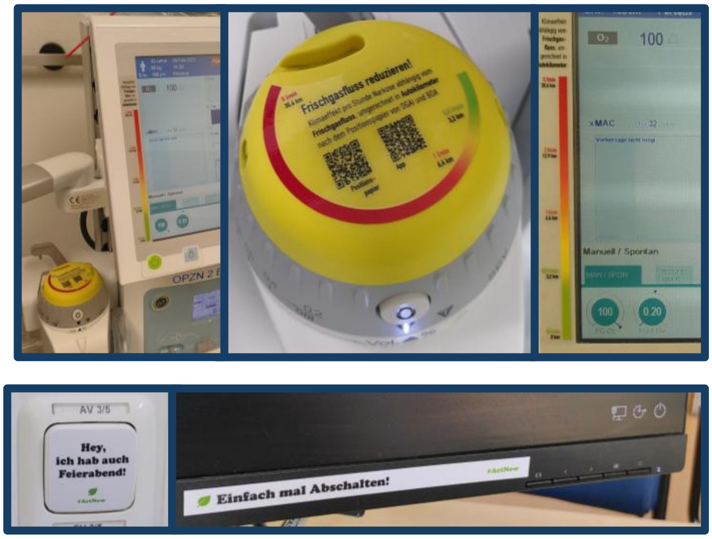

„Interventions“, is adapted from „[Supplementary Digital Material 1](https://www.minervamedica.it/en/journals/minerva-anestesiologica/article.php?cod=R02Y2024N03A0126&html=1&sdm=Supplementary%20Digital%20Material%201.pdf) - Educational material within the Provider Education and Evaluation Project“ by the authors of „Easy-to-implement educational interventions to bring climate-smart actions to daily anesthesiologic practice: a cross-sectional before and after study” published in Minerva Anestesiologica 2024 90:126–134, used under CC BY 4.0.
DOI: 10.23736/S0375-9393.23.17767-4

https://www.minervamedica.it/en/journals/minerva-anestesiologica/article.php?cod=R02Y2024N03A0126&html=1&sdm=Supplementary%20Digital%20Material%201.pdf

https://www.minervamedica.it/en/journals/minerva-anestesiologica/article.php?cod=R02Y2024N03A0126

https://www.minervamedica.it/en/journals/minerva-anestesiologica/article.php?cod=R02Y2024N03A0126https://creativecommons.org/licenses/by/4.0
